# Heterologous and endogenous *U6* snRNA promoters enable CRISPR/Cas9 mediated genome editing in *Aspergillus niger*

**DOI:** 10.1186/s40694-018-0047-4

**Published:** 2018-02-08

**Authors:** Xiaomei Zheng, Ping Zheng, Jibin Sun, Zhang Kun, Yanhe Ma

**Affiliations:** 1 Tianjin Institute of Industrial Biotechnology, Chinese Academy of Sciences, Xiqidao 32, Tianjin Airport Economic Area, Tianjin, 300308 China; 2 Key Laboratory of Systems Microbial Biotechnology, Chinese Academy of Sciences, Tianjin, 300308 China; 3 University of Chinese Academy of Sciences, Beijing, 100049 China

**Keywords:** *Aspergillus niger*, CRISPR/Cas9 system, *U6* snRNA promoters, Genome editing

## Abstract

**Background:**

*U6* promoters have been used for single guide RNA (sgRNA) transcription in the clustered regularly interspaced short palindromic repeats/CRISPR-associated protein (CRISPR/Cas9) genome editing system. However, no available *U6* promoters have been identified in *Aspergillus niger,* which is an important industrial platform for organic acid and protein production. Two CRISPR/Cas9 systems established in *A. niger* have recourse to the RNA polymerase II promoter or in vitro transcription for sgRNA synthesis, but these approaches generally increase cloning efforts and genetic manipulation. The validation of functional RNA polymerase II promoters is therefore an urgent need for *A. niger*.

**Results:**

Here, we developed a novel CRISPR/Cas9 system in *A. niger* for sgRNA expression, based on one endogenous *U6* promoter and two heterologous *U6* promoters. The three tested *U6* promoters enabled sgRNA transcription and the disruption of the polyketide synthase *albA* gene in *A. niger*. Furthermore, this system enabled highly efficient gene insertion at the targeted genome loci in *A. niger* using donor DNAs with homologous arms as short as 40-bp.

**Conclusions:**

This study demonstrated that both heterologous and endogenous *U6* promoters were functional for sgRNA expression in *A. niger*. Based on this result, a novel and simple CRISPR/Cas9 toolbox was established in *A. niger,* that will benefit future gene functional analysis and genome editing.

**Electronic supplementary material:**

The online version of this article (10.1186/s40694-018-0047-4) contains supplementary material, which is available to authorized users.

## Background

*Aspergillus niger* has attracted great attention due to its biotechnological value as a platform for producing organic acids, such as citric acid, gluconic acid and oxalic acid [1], as well as producing homologous and heterologous enzymes, including glucoamylases, amylases, acid protease, cellulase, glucose oxidase, pectinases, and xylanases [2]. *A. niger* can be used to create a promising, versatile cell factory for producing more low-priced bulk chemicals because of its remarkable unique features, including extreme acid resistance, significant robustness and powerful polymer hydrolytic enzymes. Despite its industrial importance, efficient genetic tools are generally unavailable, hampering the fundamental study and industrial improvement of this species.

The clustered regularly interspaced short palindromic repeats/CRISPR associated protein (CRISPR/Cas9) system is a powerful and revolutionary genome editing tool [3, 4]. In the CRISPR/Cas9 system, the endonuclease Cas9 is guided to a specific locus by a single guide RNA (sgRNA) where it generates a double strand break (DSB) in the genome. The DSB is usually repaired by either non-homologous end joining (NHEJ) to allow NHEJ-mediated gene disruption with base-pair insertions or deletions or homologous recombination (HR), which allows HR-mediated precise genome editing with appropriate donor DNA. The HR frequency is very low (less than 5%) in filamentous fungi [5, 6]. In traditional gene editing methods, efficiency is typically improved by increasing the homologous arm length. For example, the gene deletion efficiency was enhanced from 4 to 29%, when the homologous arm was increased from 100 to 1500-bp [6]. However, this approach had disadvantages, such a tedious donor DNA construction and onerous transformant screening. Double-stranded DNA breaks caused by Cas9 were reported to improve the HR frequency with shortened donor DNA homologous arms in *T. reesei* [7], *A. fumigatus* [8] and *P. chrysogenum* [9], whereas the CRISPR/Cas9 systems established in *A. niger* still used the donor DNA with the long homologous arms.

CRISPR/Cas9 systems have been established in *A. niger* using different strategies for sgRNA synthesis. They either depend on RNA polymerase II promoters, such as the strong promoter P*gpdA* [10] or P*mbfA* [11], or in vitro transcription is performed [12, 13]. When RNA polymerase II promoters are used, self-cleavage ribozymes, such as hepatitis delta virus (HDV) or Hammerhead (HH), are required to be added at the 5′-end and 3′-end of sgRNA, whereas the sgRNA conformation may be affected by reading-through of RNA polymerase II [10]. However, this strategy usually requires more effort when constructing sgRNA expression cassettes. As an alternative approach, Kuivanen et al. [12, 13] utilized in vitro synthesis for sgRNA expression. However, gRNA uptake and stability may influence genome editing efficiency [9]. RNA Pol III promoters for the spliceosomal *U6* snRNA have been widely used for sgRNA transcription in the CRISPR/Cas9 system. Some *U6* promoters have been used for efficient sgRNA transcription in fungi [8, 9, 14–17]. However, no *U6* promoter has been identified and validated in *A. niger*.

In this study, we aimed to establish a simple CRISPR/Cas9 system based on the *U6* promoter in *A. niger*. One endogenous *U6* promoter was identified. This endogenous promoter and two reported heterologous *U6* promoters (P*hU6* and P*yU6*) from humans and yeast were tested in *A. niger*. To enhance the simplicity of this CRISPR/Cas9 system, donor DNAs with short homologous arms (40-bp) were tested for gene insertion at DSBs induced by Cas9.

## Materials and methods

### Strains and media

*Escherichia coli* DH5α (Transgene, Beijing, China) was used for plasmid construction and cultured at 37 °C in Luria–Bertani broth containing ampicillin (100 μg/mL). The *A. niger* strains and plasmids used in this study are indicated in Additional file 1: Tables S1 and S2. *A. niger* G1 (*amdS*^−^, ∆*glaA*, and ∆*pepA*) was derived from *A. niger* NRRL3112 and presented by the Institute of Microbiology, CAS; this strain was used as the recipient strain for genome editing. *A. niger* strains were cultivated on minimal medium (MM) [18] containing 1% glucose, 70 mM NaNO_3_, 110 mM KH_2_PO_4_, 70 mM KCl, 2 mM MgSO_4_, and trace element solution or on complete medium (CM) consisting of MM supplemented with 0.5% yeast extract and 0.1% casamino acids. When using *amdS* as a selection marker, NaNO_3_ in MM was replaced by 10 mM acetamide and 15 mM caesium chloride (MMSA). For growth on solid plates, 1.5% agar was supplemented. If necessary, 150 μg/mL of hygromycin was added.

### DNA constructions

All primers used in this study are listed in Additional file 1: Table S3. The *cas9* gene from Streptococcus pyogenes was codon-optimized for expression in *A. niger*. The nuclear localization signals (NLSs) of SV40 (PKKKRKV) and nucleoplasmin (KRPAATKKAGQAKKKK) were attached into the N-termini and C-termini of codon-optimized *cas9*, which was then synthesized by Life Science Research Services Company (Genewiz, Suzhou, China). After amplification with Cas9-Fm and Cas9-Rm, *cas9* was cloned into the *Xhol*I site of the *A. niger* expressing vector pGm via the ClonExpressTM one step cloning kit (Vazyme, C113), create the Cas9 expressing plasmid pCas9. To monitor the subcellular location of Cas9, enhanced green fluorescent protein (eGFP, S65T) was fused to the C-terminus of Cas9. The (G_4_S)_3_-linker-*egfp* was amplified using pMF272 as a template with Linker-eGFP-Fm and eGFP-Rm and then was assembled into the reverse PCR product of pCAS9 (amplified using pCas9-rev-F and pCas9-rev-R) via the ClonExpress™ one step cloning kit, thus yielding pCas9GFP. The DNA sequences of codon-optimized *cas9* and *cas9gfp* are shown in Additional file 1: Table S4.

sgRNA expression constructs were synthesized containing the *Homo sapiens U6*, yeast *U6*, and *A. niger U6* promoter and sgRNA scaffolds with the *Bbs*I site inserted into the plasmid pEASY-Blunt to yield psgRNA1.0, psgRNA2.0 and psgRNA3.0, respectively. Then, targeting sgRNA constructs were built by digesting these sgRNA expression plasmids with *Bbs*I and ligating double stranded oligonucleotides with the protospacer of the *albA* gene to yield psgRNA1.1, psgRNA2.1 and psgRNA3.1. The linear sgRNA-target fragments were amplified from corresponding plasmids by PCR with M13-F and M13-R and used directly for transformation. The DNA sequences of sgRNA constructs are shown in Additional file 1: Table S5.

The donor DNA MHi-albA-hph with micro-homologous arms was synthesized by PCR amplification of the selection marker hph using the with primers MHi-albA-Fm and MHi-albA-Rm containing 40-bp homologous arms, which were homogenous to the flanking region of the *albA* sequence to be targeted. After purification, PCR products were used directly for transformation. The DNA sequences of donor DNA are shown in Additional file 1: Table S6.

### DNA transformation and analysis

*Aspergillus niger* transformation protocols, selection procedures, *A. niger* genomic DNA isolation and diagnostic PCR were performed as described in Meyer et al. [18]. The standard protocol of this novel CRISPR/Cas9 system for target gene editing is established. The construction of sgRNA with different targets and donor DNA with micro-homologous arms was followed by the co-transformation of the Cas9 expression plasmid, sgRNA, and donor DNA into the protoplasts of *A. niger* G1. The transformants were streaked on selective media at least once. After cultivating in CM rich media, genomic DNA isolation and diagnostic PCR were performed to confirm correct transformants.

For *Cas9* and *Cas9*-*eGFP* expression, 5 μg of the expression plasmid pCas9 and pCas9GFP, respectively, were transformed into *A. niger* G1 protoplasts by PEG/CaCl_2_-mediated transformation. Colonies grown on MMSA for 5 days at 30 °C were screened for the *amdS* selection marker, and diagnostic PCR was performed. The positive transformants from each construct were named *A. niger* XM1 and *A. niger* XM2, respectively (Additional file 1: Table S1).

For *albA* disruption, 5 μg of linear sgRNA PCR products and the Cas9 expression plasmid pCas9 were co-transformed into *A. niger* G1 protoplasts. Colonies grown on MMSA for 5 days at 30 °C were screened for the *amdS* selection marker, and spore phenotype statistics and sequential identification via diagnostic PCR analysis with the primers albA-g-F and albA-g-R were performed.

For *albA* gene insertion directed by donor DNA with micro-homologous arms, 5 μg linear sgRNAs PCR products, 5 μg pCas9 and 5 μg dDNA MHi-albA-hph PCR fragments were co-transformed into the protoplasts of *A. niger* G1. Transformants grown on MMSA with 100 μg/mL hygromycin B (Sigma-Aldrich, St. Louis, MO, USA) for 5 days at 30 °C were screened for *amdS* and *hph* selection markers, and spore phenotype statistics and sequential identification via diagnostic PCR and sequencing analysis with primers albA-g-F/hph-R and hph-F/albA-g-R were performed.

### Microscopic analysis of the subcellular localization of the Cas9-GFP fused protein

To analyse the subcellular localization of the Cas9-GFP fused protein, the hyphae cultivation and microscopic analysis were performed as described in Wanka et al. [19]. Briefly, two disinfected coverslips were placed onto the bottom of a small petri dish, and then 5 mL of liquid MM was supplemented with 0.003% yeast extract. After inoculation with 10^6^ spores of *A. niger*, the petri dishes were incubated for 8 h at 30 °C. After incubated with 4′, 6-diamidino-2-phenylindole (DAPI) at the final concentration of 1 mg/mL for 15 min, coverslips with adherent hyphae were placed upside down on an object slide and analysed by microscopy. Differential interference contrast (DIC) and green fluorescent images of the cells were captured with a 40× objective using a Leica DM5000B and the results were assembled in Adobe Photoshop 7.0 (Adobe, San Jose, CA).

### Quantitative reverse transcription PCR (qRT-PCR)

*Aspergillus niger* transformants were grown in triplicate in CM liquid media as described above. After 24 h of growth, mycelia were harvested, and total RNA was extracted using the RNAprep pure Plant Kit (Tiangen, Beijing). For relative RT-qPCR, total RNA was first reverse transcribed for first-strand cDNA synthesis using FastQuant RT Super Mix KR108 (Tiangen, Beijing). A 1-μL sample from the 10-fold dilution of the cDNA synthesis mix was subjected to qPCR with SuperReal PreMix Plus (SYBR Green). Real time amplification was performed using an ABI 7500 real-time PCR system. The primers sgRNA-qPCR-F and sgRNA-qPCR-R were used for the amplification of sgRNA. The relative expression level was calculated using the ΔΔCт method. 18S rRNA was used as an internal control gene and was amplified using the primers 18S rRNA-qPCR-F and 18S rRNA-qPCR-R.

## Results and discussion

### Nuclear localization of codon-optimized Cas9

**Fig. 1:**
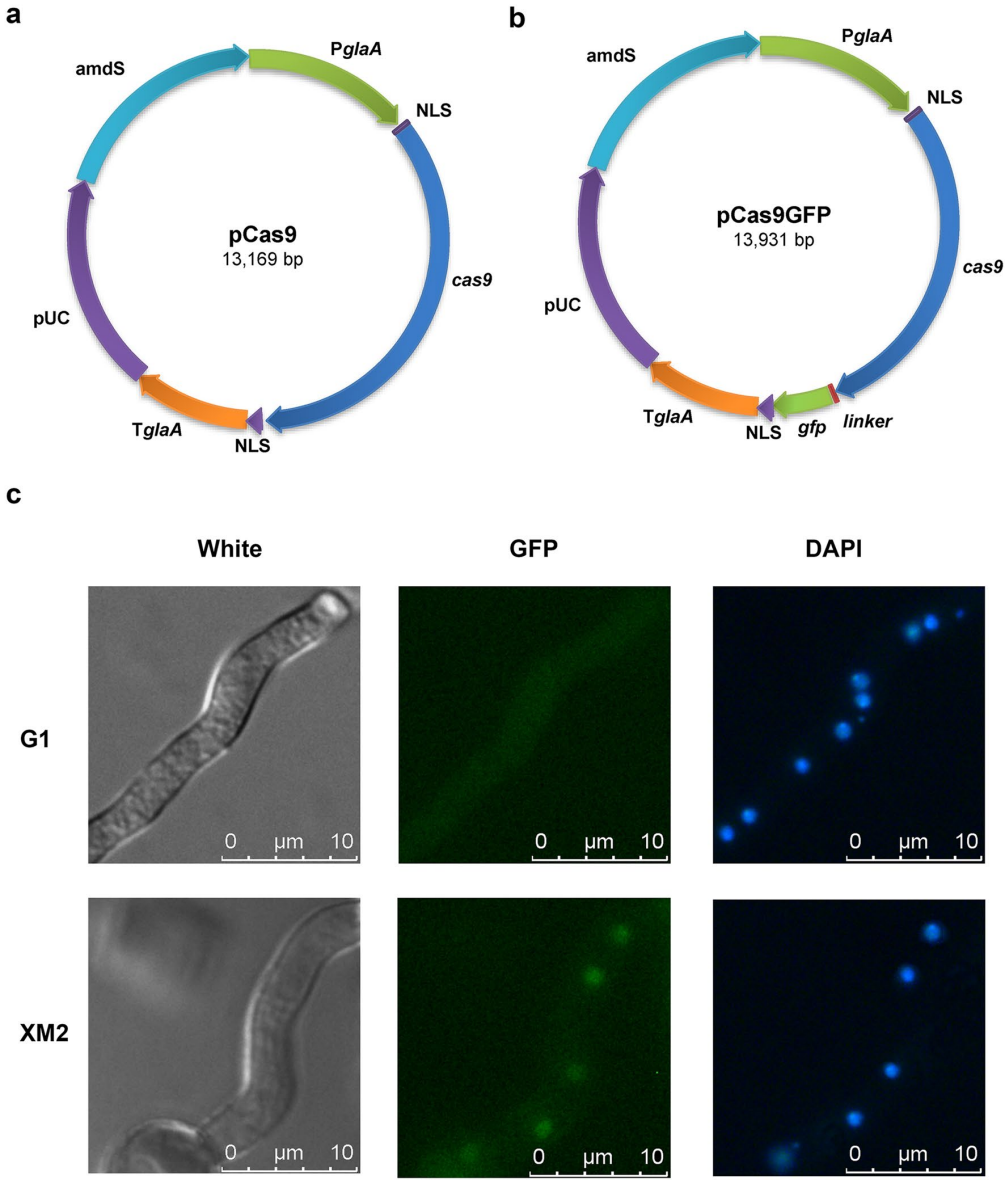
Fluorescence microscopic assessment of the localization of Cas9-GFP in *A. niger.* **a** Schematic illustration of the Cas9-expressing plasmids pCas9. **b** Schematic illustration of the Cas9-GFP fusion protein expressing plasmid pCas9GFP. **c** Fluorescent microscopic assessment of the localization of Cas9GFP in *A. niger* G1 and *A. niger* XM2

To ensure the nuclear localization of Cas9 in *A. niger*, the SV40 NLS (PKKKRKV) and the nucleoplasmin NLS (KRPAATKKAGQAKKKK) were fused onto the N-terminal and C-terminal, respectively, of Cas9 that was originally from the bacterium *Streptococcus pyogenes* but was codon-optimized for *A. niger*. Then, a Cas9 expression plasmid was constructed with the strong induced promoter P*glaA* and a universal fungal transcription terminator T*trpC* (Fig. 1a). To monitor the subcellular location of Cas9, eGFP was fused to the C-terminal of Cas9 (Fig. 1b). The plasmids pCas9 and pCas9GFP were transformed into *A. niger* G1, to generate XM1 and XM2, respectively. The localization of Cas9GFP in *A. niger* XM2 was detected by fluorescence microscopy. Compared with *A. niger* G1, the green fluorescence spots were detected in the mycelia of *A. niger* XM2, which overlapped the with the DAPI stained nuclei (Fig. 1c). This result demonstrated that Cas9 successfully localized to the nucleus with the aid of NLSs from SV40 and nucleoplasmin.

### Different *U6* promoters efficiently initiated sgRNA transcription for genomic *albA* disruption

**Fig. 2:**
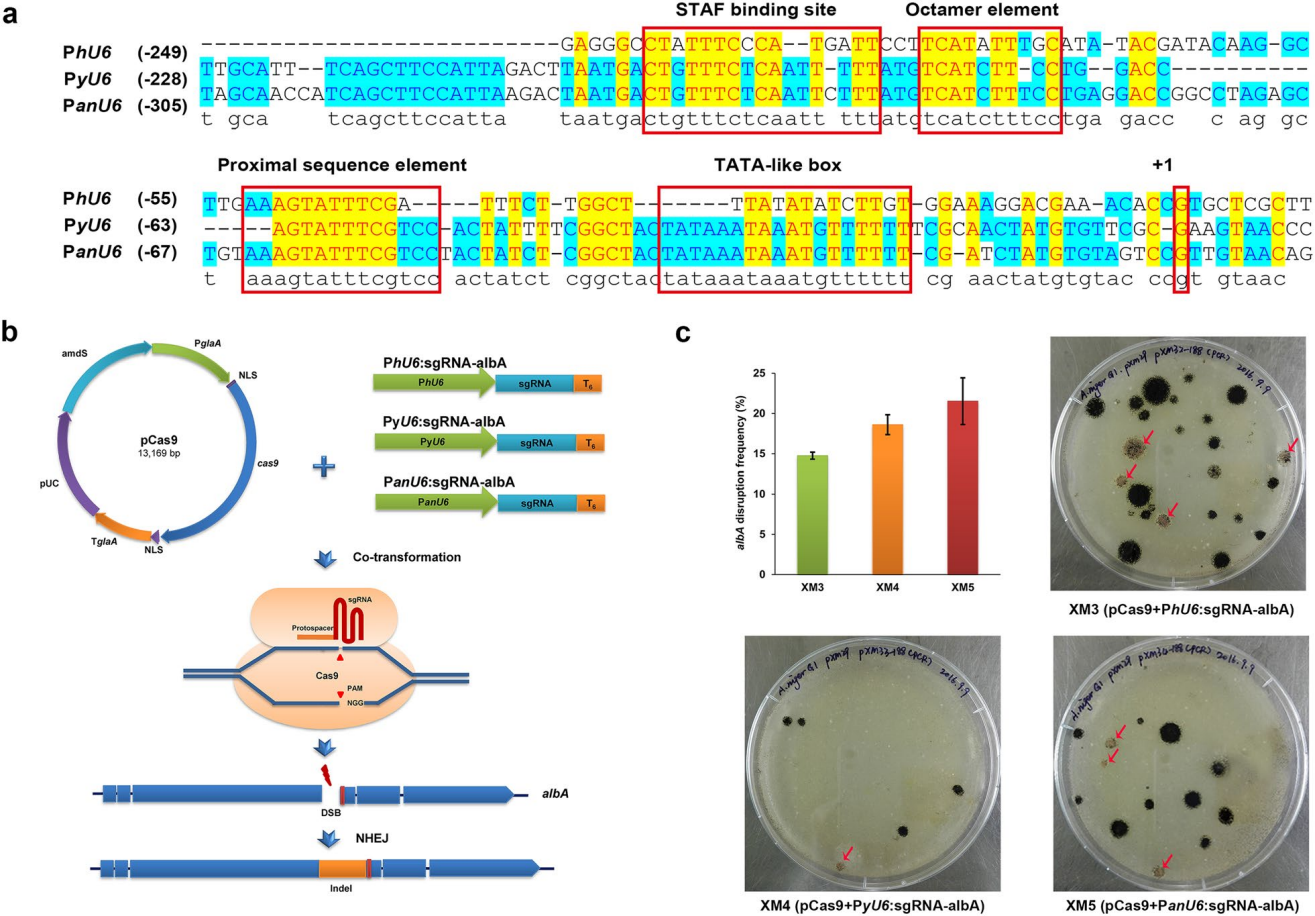
Different RNA polymerase III-based promoters for CRISPR/Cas9 systems mediated *albA* gene disruption in *A. niger.* **a** Sequence alignment of the promoter sequences of Homo sapiens RNU6-1, yeast RNU6, and *A. niger* RN*U6*. + 1 represents the transcription start; the TATA-like box and proximal and distal sequence elements are represented by a red box. **b** Schematic diagram of *albA* disruption mediated by NHEJ using the CRISPR/Cas9 system based on the *U6* promoter. *hU6* promoter represents the promoter of the human *RNU6*-*1* gene (NR_004394); the *yU6* promoter represents the promoter of the yeast *RNU6* gene (X12565.1); the *anU6* promoter represents the 412-bp upstream of *A. niger RNU6* gene (AY136823.1). T_6_ represents a string of six thymines serving as an RNA polymerase III terminator. Linear sgRNA constructs and Cas9 expression plasmid pCas9 were co-transformed into the protoplast. Without the donor DNA, the DSBs induced by Cas9 were repaired by the error-prone NHEJ system, which resulted in *albA* disruption. **c** Transformants growing on the primary transformation plates after 5 days incubation after being co-transformed with pCas9 and sgRNA expression cassettes. If *albA* was disrupted, the conidia of transformants turned pigmentless, forming albino colonies, as the red arrows indicate. The histogram shows the *albA* gene disruption efficiency of the transformants with sgRNA constructs under the control of different *U6* promoters. Bars represent the percentages of albino colonies that showed the *albA* disruption phenotype on the primary transformation plates (mean ± SD; n = 3)

To establish a simple CRISPR/Cas9 system based on the *U6* promoter in *A. niger*, we tested three *U6* promoters from different species for sgRNA expression. First, one *A. niger U6 snRNA* gene (AY136823.1) was retrieved form the NCBI GenBank database. To identify the transcription start site and promoter of this *U6 snRNA* gene, it was aligned with the *Homo sapiens RNU6* gene [20] (NR_004394) and yeast *RNU6* gene [21] (X12565.1). The 412-bp upstream of *A. niger U6 snRNA* was identified as the promoter, which showed approximately 79% identity to yeast *RNU6* promoter sequence. This *A. niger U6* promoter included some key regulatory elements, such as the TATA-like box and proximal and distal sequence elements (Fig. 2a). Interestingly, this *U6 snRNA* gene could not be amplified using the *A. niger* CBS513.88 and G1 genome as templates. Thus, the 412-bp upstream of the *A. niger U6* promoter, the *Homo sapiens U6* promoter from pX330 and the 540-bp upstream of yeast *RNU6* were synthesized and tested for sgRNA expression in *A. niger* G1.

One putative polyketide synthase (PKS) gene, *albA* (An09g05730), was chosen as the target gene, because it is involved in black spore pigmentation synthesis and its mutation leads to a visible conidial albino phenotype [10]. *albA* targeting sgRNA was constructed by the *Bbs*I digestion of sgRNA expression plasmids and ligation with double stranded oligonucleotides with a protospacer of the *albA* gene. Then, the targeting sgRNAs PCR products under the control of each *U6* promoter were co-transformed with pCas9 into the protoplasts of *A. niger* G1 (Fig. 2b). After cultivation for 5 days, conidial pigmentless colonies were brought out on the primary transformation plates. The mutants with the disrupted *albA* gene mediated by the three sgRNA constructs were designated *A. niger* XM3, XM4 and XM5. To ensure the reliability of the obtained data, we performed three transformations for each sgRNA cassette driven by these three *U6* promoters. We obtained dozens of transformants on the primary transformation plates with several albino colonies (Fig. 2c). The ratio of albino colonies was 15% (4/27 of primary transformants) for *A. niger* XM3 and 20% (1/5 of primary transformants) for *A. niger* XM4, and a slight higher ratio 23% (3/13 of primary transformants) was observed for *A. niger* XM5 (Fig. 2c). We performed diagnostic PCR for 12 purified clones isolated from the independent albino transformants to assess *albA* mutagenesis. No PCR product was obtained from these isolated albino colonies using the primers albA-g-F/albA-g-R, which spanned the PAM site, indicating that unpredicted large DNA deletion or insertion may occur in the targeted locus, as was seen in *A. fumigatus* [5, 8].

The intracellular sgRNA levels in cells with the three different *U6* promoters had obvious differences, even though the difference in the *albA* disruption ratio among the promoters was not very significant. Compared to the promoter from human P*hU6*, the endogenous promoter P*anU6* and the promoter from the yeast P*yU6* produced 43.76-fold and 6.09-fold more sgRNA, respectively (Additional file 1: Fig. S1), indicating that the *U6* endogenous promoters achieved more efficient sgRNA expression.

Our results also suggested that *U6* promoters, even from distant evolutionary species, can be used to develop the CRISPR/Cas9 system in *A. niger*. Compared to previous studies [10, 11, 13], the CRISPR/Cas9 system based on the *U6* promoter is more feasible for sgRNA expression cassette construction (Table 1) without requiring any ribozymes or in vitro synthesis of sgRNA. Moreover, it is worth mentioning that in our study, the albino colonies grew directly on primary transformation plates, rendering their isolation easier than that in previous studies reported with *Aspergilli* [8, 10]. In other studies, repeated streaking was necessary to obtain albino colonies when the target gene was disrupted by the NHEJ repair pathway. This difference between our results and those of previous studies may be caused by the genetic background of host strains, the sgRNA expression efficiency or the time of DSB generation induced by the Cas9-sgRNA complex.

**Table 1:** Comparison on CRISPR/Cas9 system for *A. niger*

Cas9 expression		sgRNA expression		Targeted gene	Donor DNA (SM/homology arm size, bp)	Gene editing efficiency	Notes	References
Promoter	Terminator	Promoter	Terminator					
P*tef1*	T*tef1*	P*gpdA*	T*trpC*	*albA*	–	Some	Requiring to add HH and HDV for processing sgRNA	[10]
P*coxA*	T*tef1*	P*mbfA*	T*trpC*	*pyrG*	–	Obtaining 25 colonies on MM with 5′-FOA	Requiring to add self-cleaving ribozymes for processing sgRNA	[11]
				*MttA*	*pyrG/*690 and 834^b^	100% (7/7)		
P*tef1*	T*tef1*	In vitro synthesis		1090836	*pyrG*/1500	28% (11/40)	3% (1/30)^a^	[12]
				1117792		100% (8/8)	43% (13/30)^a^	
				1141260		100% (8/8)	0% (0/30)^a^	
				1121140		38% (3/8)	2% (1/60)^a^	
				1146483		88% (7/8)		
				1170646		63% (5/8)		
P*glaA*	T*glaA*	P*hU6*	Ploy(T)_6_	*albA*	–	15% (4/27)	Without any selection pressure for targeted gene editing	This study
		P*yU6*			–	20% (1/5)		
		P*anU6*			–	23% (3/13)		
		P*anU6*		*albA*	*hph*/40	36% (5/14)	79% (11/14)^c^	

^a^The gene deletion efficiency without CRISPR/Cas9 system

^b^This donor DNA for gene integration was flanked by a 5′ flanking sequence of 690 bp homologous to the promoter region of the *pyrG* gene, while the 3′ flanking sequence was a mutated and truncated *pyrG* CDS of 834 bp (*pyrG*^m2, trunc^)

^c^After co-transformation of donor DNA MHi-albA-hph, sgRNA3.1 and pCas9, the outgrown albino colonies with *albA* disruption reached up to 79% (11/14). In some albino colonies, some unexpected base pair errors were mediated by NHEJ at the 5′-junction or 3′-junction of DBSs. Therefore, the precise gene integration was only 36% (5/14)

### Precise gene insertion mediated by donor DNA with short homologous arms

**Fig. 3:**
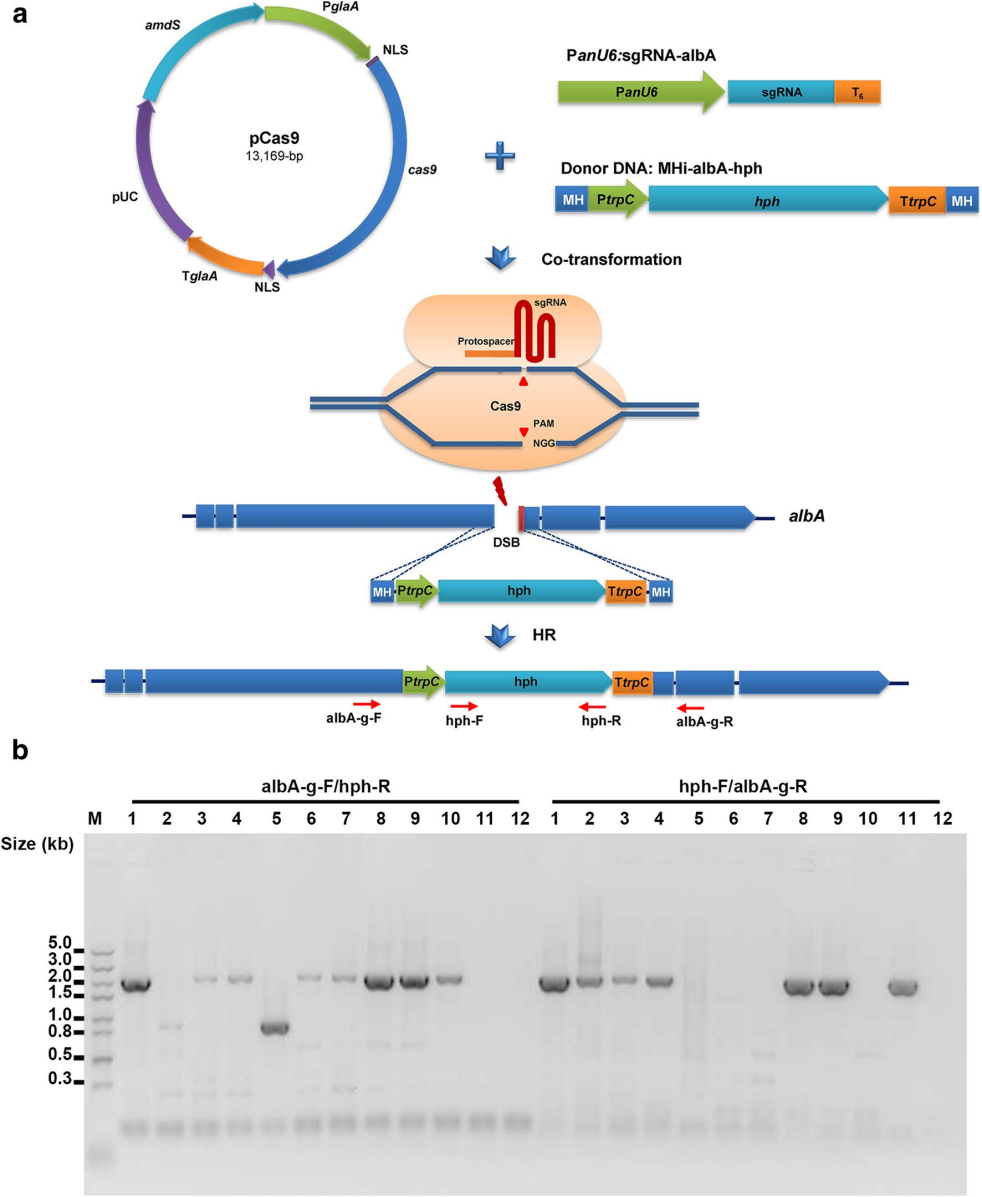
Donor DNA with 40-bp short homologous arms mediated albA gene insertion by the novel CRISPR/Cas9 system in *A. niger.* **a** Schematic diagram of *albA* gene insertion mediated by integrating the donor DNA with 40-bp micro-homologous arms. The donor DNA MHi-albA-hph was co-transformed with linear sgRNA constructs and the Cas9-expressing plasmid pCas9 into wild-type *A. niger* G1. DSBs were generated by Cas9 under the guide of the sgRNA and were then repaired by HR with the integration of MHi-albA-hph. **b** Diagnostic PCR analysis of the genetic context of DSBs in albino colonies. Correct integration of the *hph* marker at the *albA* locus: 1697 bp (albA-g-F/hph-R) and 1934 bp (hph-F/albA-g-R). M, DNA ladder; 1-11, 11 albino colonies XM6.1-6.11; 12, one black colony XM6.12 without *albA* insertion

To enhance the simplicity of this novel CRISPR/Cas9 system, the donor DNAs with short-homologous arms were used to mediate the homology-directed recombination at the double-strand DNA breaks induced by Cas9. Donor DNAs of MHi-albA-hph were designed and constructed containing 40-bp short homologous arms located next to the PAM of the *albA* protospacer. The gene insertion was accomplished by the co-transformation of pCas9, two PCR fragments of MHi-albA-hph, and sgRNA3.1 into *A. niger* G1 protoplasts (Fig. 3a). In the negative controls, when the donor DNA MHi-albA-hph was only co-transformed with pCas9 or sgRNA3.1, no albino colonies grew on the primary transformation plates (Additional file 1: Fig. S2). However, after the co-transformation of pCas9, MHi-albA-hph, and sgRNA3.1, the outgrown albino colonies accounted for 79% (11/14) of primary transformants, dramatically increasing the *albA* gene disruption efficiency (Additional file 1: Fig. S2). This result indicated that the CRISPR/Cas9 system based on the *U6* promoter improved the gene editing efficiency and allowed the usage of donor DNA containing short homologous arms. Kuivanen et al. [12] also found that the gene editing efficiency was significantly increased with the assistance of the CRISPR/Cas9 system (Table 1).

The genetic context for the DSBs in 11 albino colonies and one black colony as a negative control were determined via PCR and DNA sequencing using two pairs of primers (Fig. 3b and Additional file 1: Fig. S3). Among 11 albino colonies, the expected PCR products were amplified in five colonies, i.e., XM6.1, 6.3, 6.4, 6.8, and 6.9, suggesting they carried the correct *hph* insertion at both expected cleavage sites (Fig. 3b, *lanes* 1, 3, 4, 8, and 9; Additional file 1: Fig. S3). For the other albino colonies, only one correct PCR product was amplified, indicating that the *hph* cassette was inserted at only the 5′-junction or 3′-junction (Fig. 3b, *lanes* 6, 7, 10, and 11; Additional file 1: Fig. S3). For the albino colony XM6.5, only one smaller PCR product was detected, indicating an 800-bp-deletion when the *hph* cassette was inserted at the 3′-junction (Fig. 3b, *lane* 5; Additional file 1: Fig. S3). The albino colony XM6.2 had a mixed genotype, similar to XM6.5 at the 3′-junction lane 5 and correct insertion at the 5′-junction (Fig. 3b, *lane* 2; Additional file 1: Fig. S3). Zhang et al. [8] found that donor DNAs with 39-bp or 28-bp homologous arms were sufficient to precisely induce mutagenesis in *A. fumigatus* in a NHEJ system-independent manner. These differences could be caused by the high activity of error-prone NHEJ or by micro-homology-mediated end joining (MMEJ) [22] in the *A. niger* wild type strain.

Clearly, we have confirmed that, combined with the CRISPR/Cas9 system, short homologous arms as short 40-bp are sufficient for mediating targeted gene insertion, which facilitates the construction of donor DNA in *A. niger*. Moreover, due to the complicated genomic repair outcomes at the DSBs in wild-type strains, it is recommended that more attention should be paid to mutant genotypes for precise editing.

## Conclusions

In conclusion, we established a simple CRISPR/Cas9 system based on the *U6* promoter in *A. niger*. Two heterologous (P*hU6* and P*yU6*) and one endogenous *U6* promoter were capable of driving the transcription of sgRNA, which guided Cas9 to the target site for generating DSBs. Donor DNAs with short homologous arms (40-bp) were sufficient for insertion at DSBs induced by Cas9, simplifying and increasing the convenience of genetic manipulation in *A. niger*.

## Additional file

**Additional file 1. Figure S1.** qPCR results of sgRNA expression levels from each promoter. Total RNA was isolated, converted to cDNA, and sgRNA expression level was quantified. sgRNA expression levels were normalized to the amount of sgRNA generated by P*hU6* promoter. 18S rRNA was used as internal control. Bars represent the fold change of sgRNA level under the control of different U6 promoters (mean ± SD; n = 3).** Figure S2.** Transformants with* albA* disruption by inserted the donor DNA with short homologous arms. Transformants XM6 grew on the primary transformation plates after co-transformed pCas9, sgRNA3.1 and donor DNA MHi-albA-hph. Transformants NC1 grew on the primary transformation plates after only co-transformed pCas9 and donor DNA MHi-albA-hph. Transformants NC2 grew on the primary transformation plates after only co-transformed sgRNA3.1 and donor DNA MHi-albA-hph.** Figure S3.** DNA sequencing analyses for genetic context at the DSBs in albA gene inserted transformants XM6. DNA sequencing results of PCR products amplified by albA-g-F/hph-R (a) and hph-F/albA-g-R (b) using the genomic DNA of albino colonies XM6 as templates. The red letters represent the protospacer sgRNA-albA1, and the yellow shaded red letters represent the PAM site. The green letters represent the to-be-inserted hph cassette, and blue letters represent the homology arms in the donor DNA MHi-albA-hph. XM6.1-6.11 represent the selected albino colonies.** Table S1.** A. niger strains used in this study.** Table S2.** Plasmids used in this study. **Table S3.** Primers used in this study. Restriction sites are underlined. Fm represents forward primer with modification and Rm represents reverse primer with modification. The modified additional sequences were represented in lowercase letters.** Table S4.** DNA sequences of codon optimized* cas9* used in this study. Black letters indicate the codon-optimized* cas9* gene. Purple letter indicate the NLS sequences of SV40 at 5’-termini and nucleoplasmin at 3’-termini. Green letters indicate the* gfp* (S65T) gene from pMF272. Orange letters indicate the (G_4_S)_3_ linker sequence.** Table S5.** DNA sequences of sgRNA constructs used in this study. Green letters indicate the promoter region for sgRNA expression. Orange letters indicate the transcription start of* U6* promoters. Blue letters indicate the sgRNA scaffold. Blue underlined letters indicate* BbsI* restriction sites. Red letters indicate the terminator of* RNU6* gene. Red underlined letters indicate genetic targets.** Table S6.** DNA sequences of donor DNA used in this study. Blue letters indicate the homogenous arms located at the 5’ and 3’ flanking region of the genetic target sites. Black lowercase letters indicate the selection marker cassettes.

## Abbreviations

CRISPR: clustered regularly interspaced short palindromic repeats
Cas9: CRISPR associated protein 9
sgRNA: single guide RNA
DSB: double-strand DNA break
PAM: protospacer adjacent motif
HR: homologous recombination
NHEJ: non-homologous end joining
