# Supplementary material for: Heterologous and endogenous U6 snRNA promoters enable CRISPR/Cas9 mediated genome editing in Aspergillus niger
Source: Fungal Biol Biotechnol. 2018 Feb 8;5:2. doi: 10.1186/s40694-018-0047-4 (PMC5806293; doi:10.1186/s40694-018-0047-4)
Supplement: Supplementary file 1 — Additional file 1. Figure S1. qPCR results of sgRNA expression levels from each promoter. Total RNA was isolated, converted to cDNA, and sgRNA expression level was quantified. sgRNA expression levels were normalized to the amount of sgRNA generated by PhU6 promoter. 18S rRNA was used as internal control. Bars represent the fold change of sgRNA level under the control of different U6 promoters (mean ± SD; n = 3). Figure S2. Transformants with albA disruption by inserted the donor DNA with short homologous arms. Transformants XM6 grew on the primary transformation plates after co-transformed pCas9, sgRNA3.1 and donor DNA MHi-albA-hph. Transformants NC1 grew on the primary transformation plates after only co-transformed pCas9 and donor DNA MHi-albA-hph. Transformants NC2 grew on the primary transformation plates after only co-transformed sgRNA3.1 and donor DNA MHi-albA-hph. Figure S3. DNA sequencing analyses for genetic context at the DSBs in albA gene inserted transformants XM6. DNA sequencing results of PCR products amplified by albA-g-F/hph-R (a) and hph-F/albA-g-R (b) using the genomic DNA of albino colonies XM6 as templates. The red letters represent the protospacer sgRNA-albA1, and the yellow shaded red letters represent the PAM site. The green letters represent the to-be-inserted hph cassette, and blue letters represent the homology arms in the donor DNA MHi-albA-hph. XM6.1-6.11 represent the selected albino colonies. Table S1. A. niger strains used in this study. Table S2. Plasmids used in this study. Table S3. Primers used in this study. Restriction sites are underlined. Fm represents forward primer with modification and Rm represents reverse primer with modification. The modified additional sequences were represented in lowercase letters. Table S4. DNA sequences of codon optimized cas9 used in this study. Black letters indicate the codon-optimized cas9 gene. Purple letter indicate the NLS sequences of SV40 at 5’-termini and nucleoplasmin at 3’-termini. Green [file 40694_2018_47_MOESM1_ESM.docx]

**Supporting Information**

**Heterologous and endogenous *U6* snRNA promoters enabled CRISPR/Cas9 mediated genome editing in *Aspergillus niger***

Xiaomei Zheng^1, 2^ Ping Zheng^1, 2*^ Jibin Sun^1, 2*^ Zhang Kun^1, 2, 3^ Yanhe Ma^1^

^1^ Tianjin Institute of Industrial Biotechnology, Chinese Academy of Sciences, Tianjin, 300308 China

^2^ Key Laboratory of Systems Microbial Biotechnology, Chinese Academy of Sciences, Tianjin, 300308 China

^3^ University of Chinese Academy of Sciences, Beijing, 100049 China

#### Contents

#### Fig. S1 qPCR results of sgRNA expression levels from each promoter.

#### Fig. S2 Transformants with *albA* disruption by inserted the donor DNA with short homologous arms.

## Fig. S3 DNA sequencing analyses for genetic context at the DSBs in *albA* gene inserted transformants XM6

## Table S1 *A. niger* strains used in this study

## Table S2 Plasmids used in this study

## Table S3 Primers used in this study

## Table S4 DNA sequences of codon optimized *cas9* used in this study

## Table S5 DNA sequences of sgRNA constructs used in this study

## Table S6 DNA sequences of donor DNA used in this study

#### **Fig. S1**


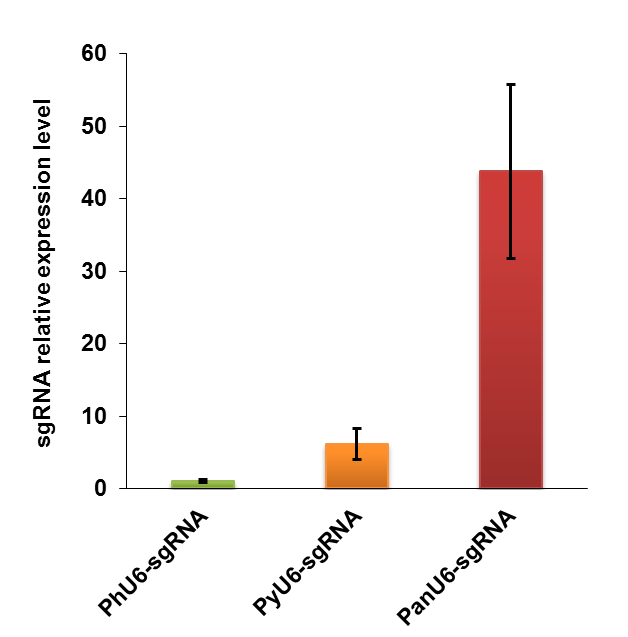


**Fig. S1 qPCR results of sgRNA expression levels from each promoter.**

Total RNA was isolated, converted to cDNA, and sgRNA expression level was quantified. sgRNA expression levels were normalized to the amount of sgRNA generated by *PhU6* promoter. 18S rRNA was used as internal control. Bars represent the fold change of sgRNA level under the control of different *U6* promoters (mean ± SD; n = 3).

#### **Fig. S2**

**Fig. S2 Transformants with *albA* disruption by inserted the donor DNA with short homologous arms.**

Transformants XM6 grew on the primary transformation plates after co-transformed pCas9, sgRNA3.1 and donor DNA MHi-albA-hph. Transformants NC1 grew on the primary transformation plates after only co-transformed pCas9 and donor DNA MHi-albA-hph. Transformants NC2 grew on the primary transformation plates after only co-transformed sgRNA3.1 and donor DNA MHi-albA-hph.

#### **Fig. S3**

**Fig. S3 DNA sequencing analyses for genetic context at the DSBs in *albA* gene inserted transformants XM6**

DNA sequencing results of PCR products amplified by albA-g-F/hph-R (a) and hph-F/albA-g-R (b) using the genomic DNA of albino colonies XM6 as templates. The red letters represent the protospacer sgRNA-albA1, and the yellow shaded red letters represent the PAM site. The green letters represent the to-be-inserted *hph* cassette, and blue letters represent the homology arms in the donor DNA MHi-albA-hph. XM6.1-6.11 represent the selected albino colonies.

**Table S1 *A. niger* strains used in this study**

| Strain | Genotype | Description | Source |
| --- | --- | --- | --- |
| G1 | *amdS^-^*, ∆*glaA*, ∆*pepA* | A derivative of NRRL3112 | Lab store |
| XM1 | *Cas9^+^*, *amdS^+^*, ∆*glaA*, ∆*pepA* | G1 derivative with codon optimized Cas9 cassette | This study |
| XM2 | *Cas9-gfp^+^*, *amdS^+^*, ∆*glaA*, ∆*pepA* | G1 derivative with codon optimized Cas9-GFP cassette | This study |
| XM3 | *Cas9^+^*, *amdS^+^*, ∆*glaA*, ∆*pepA*, *albA*^off^ | G1 derivative with albA disruption via targeting sgRNA driven by *homo* *U6* promoter | This study |
| XM4 | *Cas9^+^*, *amdS^+^*, ∆*glaA*, ∆*pepA*, *albA*^off^ | G1 derivative with albA disruption via targeting sgRNA driven by *yeast U6* promoter | This study |
| XM5 | *Cas9^+^*, *amdS^+^*, ∆*glaA*, ∆*pepA*, *albA*^off^ | G1 derivative with albA disruption via targeting sgRNA driven by *A. niger* *U6* promoter | This study |
| XM6 | *Cas9^+^*, *amdS^+^*, ∆*glaA*, ∆*pepA*, ∆*albA::hyh* | G1 derivative with insertion of selection marker *hph* cassette in *albA* | This study |

**Table S2 Plasmids used in this study**

| Plasmid | Expression cassette | sgRNA Target | Backbone | Usage |
| --- | --- | --- | --- | --- |
| pCas9 | P*glaA*:nls-*cas9*-nls:T*glaA* | N/A | pGm | Cas9 expression |
| pCas9GFP | P*glaA*:nls-*cas9gfp*-nls:T*glaA* | N/A | pGm | Cas9 expression |
| psgRNA1.0 | P*hU6*:sgRNA:T*poly(T)_6_* | empty | pEASY-Blunt | sgRNA contruct |
| psgRNA2.0 | P*yU6*:sgRNA:T*poly(T)_6_* | empty | pEASY-Blunt | sgRNA contruct |
| psgRNA3.0 | P*anU6*:sgRNA:T*poly(T)_6_* | empty | pEASY-Blunt | sgRNA contruct |
| psgRNA1.1 | P*hU6*:sgRNA:T*poly(T)_6_* | *albA* | pEASY-Blunt | Gene disruption |
| psgRNA2.1 | P*yU6*:sgRNA:T*poly(T)_6_* | *albA* | pEASY-Blunt | Gene disruption |
| psgRNA3.1 | P*anU6*:sgRNA:T*poly(T)_6_* | *albA* | pEASY-Blunt | Gene disruption |

**Table S3 Primers used in this study**

| Primer name | | Primer Sequence (5' to 3') | |
| --- | --- | --- | --- |
| \| **Primers to construct Cas9 expressing plamids** \| \| --- \| | | | |
| Cas9-Fm | | catccccagcatcattacacctcgagACCATGCCAAAGAAGAAGCGGAAGG | |
| Cas9-Rm | | tattaattaaggccggccttaagctcgagTCACTTCTTCTTCTTAGCCTGG | |
| pCas9-rev-F | AAGCGCCCCGCCGCCACCAAG | |  |
| pCas9-rev-R | GTCGCCGCCGAGCTGGGAGAG | |  |
| Linker-eGFP-Fm | ctctcccagctcggcggcgac*ggcggtggcggatccggcggtggcggttccggcggtggcggttcc*  ATGGTGAGCAAGGGCGAGG | |  |
| eGFP-Rm | cttggtggcggcggggcgcttCTTGTACAGCTCGTCCATGC | |  |
| pGm-F | | CGGAGATTCGTCGCCTAATGTC | |
| pGm-R | | CCGTCGGTCGCAATACAATCAC | |
| \| **Primers to construct sgRNA expressing cassette** \| \| --- \| | | | |
| PhU6-F | | GAGGGCCTATTTCCCATGATTC | |
| PyU6-F | | GATCGATAATTCTCCATAATAG | |
| PanU6-F | | CCCAAGCTTGATCGATAATTC | |
| sgRNA-R | | AAAAAAGCACCGACTCGGTGCCA | |
| M13F | TAAAACGACGGCCAG | |  |
| M13R | CAGGAAACAGCTATGAC | |  |
| \| **Primers to construct targeting sgRNA** \| \| --- \| | | | |
| sgRNA-albA-F | | caccAGTGGGATCTCAAGAACTAC | |
| sgRNA-albA-R | | aaacGTAGTTCTTGAGATCCCACT | |
| \| **Primers to construct donor DNAs with micro-homologue arms** \| \| --- \| | | | |
| MHAi-albA1-Fm | | cctccgcctcccagcctacaagtgggatctcaagaactacGACGTTAACTGATATTGAAGGAGC | |
| MHAi-albA1-Rm | | gagcgcccttgctcaggcagaagttgttggtatagggaatAACCCAGGGGCTGGTGACGG | |
| Primers to diagnostic PCR of gene editing transformants | | | |
| albA-g-F | | CGAGTTGGCTCAGAAATGCTC | |
| albA-g-R | | CGGGATCAGCAATGTCGTTC | |
| hph-F | | ATGAAAAAGCCTGAACTC | |
| hph-R | | CTATTCCTTTGCCCTCGGACGAG | |
| Primers to qPCR for detecting the sgRNA expression level | | | |
| 18S rRNA-qPCR-F | | AATAAGGATTGACAGATTGAGAG | |
| 18S rRNA-qPCR-R | | CACTTCCATCGGCTTGAG | |
| sgRNA-qPCR-F | | GTTTTAGAGCTAGAAATAGCAAG | |
| sgRNA-qPCR-R | | AAAAGCACCGACTCGGT | |

Notes: Restriction sites are underlined. Fm represents forward primer with modification and Rm represents reverse primer with modification. The modified additional sequences were represented in lowercase letters.

## Table S4 DNA sequences of codon optimized *cas9* used in this study

| Gene | DNA Sequence (5’ to 3’) |
| --- | --- |
| codon optimized *cas9* | ATGCCAAAGAAGAAGCGGAAGGTCATGGATAAGAAGTACTCCATCGGCCTCGACATCGGCACCAACTCCGTCGGCTGGGCCGTCATCACCGATGAGTACAAGGTCCCTTCCAAGAAGTTCAAGGTCCTCGGCAACACCGATCGCCATTCCATCAAGAAGAACCTGATCGGCGCCCTCCTGTTCGATTCCGGCGAAACCGCCGAGGCCACCCGCCTTAAACGCACCGCCCGTCGCCGCTACACCCGCCGCAAGAACCGCATCTGCTACCTCCAAGAAATCTTCTCCAACGAGATGGCCAAGGTCGATGATAGCTTCTTCCACCGCCTCGAAGAGTCCTTCCTGGTCGAAGAGGATAAGAAGCACGAGCGCCATCCTATCTTCGGCAACATCGTCGATGAGGTCGCCTACCATGAGAAGTACCCTACCATCTACCATCTCCGCAAGAAGCTCGTCGATTCCACCGATAAGGCCGATCTCCGCCTCATCTACCTCGCCCTCGCCCATATGATCAAGTTCCGCGGCCATTTCCTCATCGAGGGCGATCTCAACCCTGATAACTCCGATGTCGATAAGCTGTTCATCCAGCTCGTCCAGACCTACAACCAGCTGTTCGAGGAAAACCCTATCAACGCCTCCGGCGTCGATGCCAAGGCCATCCTCTCCGCTCGCCTCTCCAAGTCTCGCCGCCTTGAGAACCTTATCGCCCAGCTCCCTGGCGAGAAGAAGAACGGCCTCTTCGGCAACCTGATCGCCCTCTCCCTCGGCCTCACCCCTAACTTCAAGTCCAACTTCGATCTCGCCGAGGATGCCAAGCTCCAGCTCTCCAAGGATACCTACGATGATGATCTCGATAACCTCCTCGCCCAGATCGGCGATCAGTACGCCGATCTGTTCCTCGCCGCCAAGAACCTCTCCGATGCCATCCTCCTCTCCGACATCCTCCGCGTCAACACCGAGATCACCAAGGCCCCTCTGTCCGCCTCCATGATCAAGCGCTACGATGAGCATCATCAGGACCTCACCCTGCTCAAGGCCCTCGTCCGCCAGCAGCTCCCTGAGAAGTACAAAGAGATTTTCTTCGATCAGTCCAAGAACGGCTACGCCGGCTACATCGATGGCGGCGCTTCCCAAGAAGAGTTCTACAAGTTCATCAAGCCTATCCTTGAGAAGATGGATGGCACCGAGGAACTCCTCGTCAAGCTCAACCGCGAGGACCTCCTCCGCAAGCAGCGCACCTTCGATAACGGCTCCATCCCTCATCAAATCCATCTCGGCGAGCTGCATGCCATCTTGCGCCGCCAAGAGGATTTCTACCCATTCCTCAAGGATAACCGCGAGAAGATCGAAAAGATTCTCACCTTCCGCATCCCTTACTACGTCGGCCCTCTCGCTCGCGGCAACTCCCGCTTCGCCTGGATGACCCGCAAGTCCGAGGAAACCATCACCCCTTGGAACTTCGAGGAAGTCGTCGATAAGGGCGCCTCCGCCCAGTCCTTCATCGAGCGCATGACCAACTTCGATAAGAACCTCCCTAACGAGAAGGTCCTCCCTAAGCACTCCCTGCTCTACGAGTACTTCACCGTCTACAACGAGCTGACCAAGGTCAAGTACGTCACCGAGGGTATGCGCAAGCCTGCCTTCCTGTCCGGCGAGCAGAAGAAGGCCATCGTCGATCTGCTGTTCAAGACCAACCGCAAGGTCACCGTCAAGCAGCTCAAAGAGGATTACTTCAAGAAAATCGAGTGCTTCGATTCCGTCGAGATCAGCGGCGTCGAGGACCGCTTCAACGCCTCCCTCGGAACCTACCATGATCTCCTCAAGATTATCAAGGATAAGGATTTCCTCGACAACGAGGAAAACGAGGACATCCTTGAGGACATCGTCCTCACCCTCACCCTCTTCGAGGACCGCGAAATGATCGAGGAACGCCTCAAGACCTACGCCCATCTCTTCGATGATAAGGTCATGAAGCAGCTCAAGCGCCGTCGCTACACCGGCTGGGGTCGCCTCTCCCGCAAGCTCATCAACGGCATCCGCGATAAGCAGTCCGGCAAGACTATCCTCGATTTCCTCAAGTCCGATGGCTTCGCCAACCGCAACTTCATGCAGCTCATCCATGATGATTCCCTCACCTTCAAAGAGGACATCCAGAAGGCCCAGGTCAGCGGCCAGGGCGATTCCCTCCATGAGCATATCGCCAACCTCGCCGGCTCCCCTGCCATCAAGAAGGGCATCCTCCAGACCGTCAAGGTCGTCGATGAGCTGGTCAAGGTCATGGGCCGCCATAAGCCTGAGAACATCGTCATCGAGATGGCCCGCGAGAACCAGACCACCCAGAAGGGCCAGAAGAACTCCCGCGAGCGCATGAAGCGCATCGAGGAAGGCATCAAAGAGCTGGGCAGCCAAATCCTCAAAGAGCATCCTGTCGAGAACACCCAGCTCCAGAACGAGAAGCTCTACCTCTACTACCTCCAGAACGGCCGCGATATGTACGTCGATCAAGAGCTGGACATCAACCGCCTCTCCGATTACGATGTCGATCATATCGTCCCTCAGTCCTTCCTGAAGGATGATTCCATCGATAACAAGGTCCTCACCCGCTCCGATAAGAACCGCGGCAAGTCCGATAACGTCCCTTCCGAAGAGGTCGTCAAGAAGATGAAGAACTACTGGCGCCAGCTCCTCAACGCCAAGCTCATCACCCAGCGCAAGTTCGATAACCTCACCAAGGCCGAGCGCGGTGGCCTCTCCGAGCTGGATAAGGCCGGCTTCATCAAGCGCCAGCTCGTCGAAACCCGCCAGATCACCAAGCACGTCGCCCAAATCCTCGATTCCCGCATGAACACCAAGTACGATGAGAACGATAAGCTCATCCGCGAAGTCAAGGTCATCACCCTCAAGTCCAAGCTCGTCAGCGATTTCCGCAAGGATTTCCAGTTCTACAAGGTCCGCGAGATCAACAACTACCATCATGCCCATGATGCCTACCTCAACGCCGTCGTCGGCACCGCCCTCATCAAGAAGTACCCCAAGCTCGAATCCGAGTTCGTCTACGGTGATTACAAGGTCTACGATGTCCGCAAGATGATCGCCAAGTCCGAGCAAGAGATCGGCAAGGCTACCGCCAAGTACTTCTTCTACTCCAACATCATGAATTTCTTCAAGACCGAAATCACCCTCGCCAACGGCGAAATCCGCAAGCGCCCTCTCATCGAGACTAACGGCGAGACTGGCGAGATCGTCTGGGATAAGGGCCGCGATTTCGCCACCGTCCGCAAGGTCCTCTCCATGCCTCAGGTCAACATCGTCAAGAAAACCGAGGTCCAGACCGGCGGCTTCTCCAAAGAGTCCATCCTCCCCAAGCGCAACTCCGATAAGCTGATCGCCCGCAAGAAGGATTGGGACCCTAAGAAGTACGGCGGCTTCGATTCCCCTACCGTCGCCTACTCCGTCCTCGTCGTCGCCAAGGTCGAGAAGGGCAAGTCCAAGAAGCTCAAGTCCGTCAAAGAGCTGCTCGGCATCACTATTATGGAACGCTCCAGCTTCGAGAAGAACCCTATCGATTTCCTTGAGGCCAAGGGCTACAAAGAGGTCAAGAAGGACCTCATCATCAAGCTCCCCAAGTACTCCCTGTTCGAGCTTGAGAACGGCCGCAAGCGCATGCTCGCCTCCGCCGGTGAGCTTCAGAAGGGCAACGAGCTGGCCCTGCCTTCCAAGTACGTCAACTTCCTCTACCTCGCCTCCCATTACGAGAAGCTCAAGGGCTCCCCTGAGGATAACGAGCAGAAGCAGCTGTTCGTCGAGCAGCATAAGCACTACCTCGATGAGATCATCGAGCAGATCAGCGAGTTCTCCAAGCGCGTCATCCTCGCCGATGCCAACCTCGATAAGGTCCTGTCCGCCTACAACAAGCACCGCGATAAGCCTATCCGCGAGCAGGCCGAGAACATCATCCATCTCTTCACCCTCACCAACCTCGGTGCCCCTGCCGCCTTCAAGTACTTCGATACCACCATCGATCGCAAGCGCTACACCTCCACCAAAGAGGTCCTGGACGCCACCCTCATCCATCAGTCCATCACCGGCCTCTACGAAACCCGCATCGATCTCTCCCAGCTCGGCGGCGACAAGCGCCCCGCCGCCACCAAGAAGGCCGGCCAGGCTAAGAAGAAGAAGTGA |
| *cas9gfp* | ATGCCAAAGAAGAAGCGGAAGGTCATGGATAAGAAGTACTCCATCGGCCTCGACATCGGCACCAACTCCGTCGGCTGGGCCGTCATCACCGATGAGTACAAGGTCCCTTCCAAGAAGTTCAAGGTCCTCGGCAACACCGATCGCCATTCCATCAAGAAGAACCTGATCGGCGCCCTCCTGTTCGATTCCGGCGAAACCGCCGAGGCCACCCGCCTTAAACGCACCGCCCGTCGCCGCTACACCCGCCGCAAGAACCGCATCTGCTACCTCCAAGAAATCTTCTCCAACGAGATGGCCAAGGTCGATGATAGCTTCTTCCACCGCCTCGAAGAGTCCTTCCTGGTCGAAGAGGATAAGAAGCACGAGCGCCATCCTATCTTCGGCAACATCGTCGATGAGGTCGCCTACCATGAGAAGTACCCTACCATCTACCATCTCCGCAAGAAGCTCGTCGATTCCACCGATAAGGCCGATCTCCGCCTCATCTACCTCGCCCTCGCCCATATGATCAAGTTCCGCGGCCATTTCCTCATCGAGGGCGATCTCAACCCTGATAACTCCGATGTCGATAAGCTGTTCATCCAGCTCGTCCAGACCTACAACCAGCTGTTCGAGGAAAACCCTATCAACGCCTCCGGCGTCGATGCCAAGGCCATCCTCTCCGCTCGCCTCTCCAAGTCTCGCCGCCTTGAGAACCTTATCGCCCAGCTCCCTGGCGAGAAGAAGAACGGCCTCTTCGGCAACCTGATCGCCCTCTCCCTCGGCCTCACCCCTAACTTCAAGTCCAACTTCGATCTCGCCGAGGATGCCAAGCTCCAGCTCTCCAAGGATACCTACGATGATGATCTCGATAACCTCCTCGCCCAGATCGGCGATCAGTACGCCGATCTGTTCCTCGCCGCCAAGAACCTCTCCGATGCCATCCTCCTCTCCGACATCCTCCGCGTCAACACCGAGATCACCAAGGCCCCTCTGTCCGCCTCCATGATCAAGCGCTACGATGAGCATCATCAGGACCTCACCCTGCTCAAGGCCCTCGTCCGCCAGCAGCTCCCTGAGAAGTACAAAGAGATTTTCTTCGATCAGTCCAAGAACGGCTACGCCGGCTACATCGATGGCGGCGCTTCCCAAGAAGAGTTCTACAAGTTCATCAAGCCTATCCTTGAGAAGATGGATGGCACCGAGGAACTCCTCGTCAAGCTCAACCGCGAGGACCTCCTCCGCAAGCAGCGCACCTTCGATAACGGCTCCATCCCTCATCAAATCCATCTCGGCGAGCTGCATGCCATCTTGCGCCGCCAAGAGGATTTCTACCCATTCCTCAAGGATAACCGCGAGAAGATCGAAAAGATTCTCACCTTCCGCATCCCTTACTACGTCGGCCCTCTCGCTCGCGGCAACTCCCGCTTCGCCTGGATGACCCGCAAGTCCGAGGAAACCATCACCCCTTGGAACTTCGAGGAAGTCGTCGATAAGGGCGCCTCCGCCCAGTCCTTCATCGAGCGCATGACCAACTTCGATAAGAACCTCCCTAACGAGAAGGTCCTCCCTAAGCACTCCCTGCTCTACGAGTACTTCACCGTCTACAACGAGCTGACCAAGGTCAAGTACGTCACCGAGGGTATGCGCAAGCCTGCCTTCCTGTCCGGCGAGCAGAAGAAGGCCATCGTCGATCTGCTGTTCAAGACCAACCGCAAGGTCACCGTCAAGCAGCTCAAAGAGGATTACTTCAAGAAAATCGAGTGCTTCGATTCCGTCGAGATCAGCGGCGTCGAGGACCGCTTCAACGCCTCCCTCGGAACCTACCATGATCTCCTCAAGATTATCAAGGATAAGGATTTCCTCGACAACGAGGAAAACGAGGACATCCTTGAGGACATCGTCCTCACCCTCACCCTCTTCGAGGACCGCGAAATGATCGAGGAACGCCTCAAGACCTACGCCCATCTCTTCGATGATAAGGTCATGAAGCAGCTCAAGCGCCGTCGCTACACCGGCTGGGGTCGCCTCTCCCGCAAGCTCATCAACGGCATCCGCGATAAGCAGTCCGGCAAGACTATCCTCGATTTCCTCAAGTCCGATGGCTTCGCCAACCGCAACTTCATGCAGCTCATCCATGATGATTCCCTCACCTTCAAAGAGGACATCCAGAAGGCCCAGGTCAGCGGCCAGGGCGATTCCCTCCATGAGCATATCGCCAACCTCGCCGGCTCCCCTGCCATCAAGAAGGGCATCCTCCAGACCGTCAAGGTCGTCGATGAGCTGGTCAAGGTCATGGGCCGCCATAAGCCTGAGAACATCGTCATCGAGATGGCCCGCGAGAACCAGACCACCCAGAAGGGCCAGAAGAACTCCCGCGAGCGCATGAAGCGCATCGAGGAAGGCATCAAAGAGCTGGGCAGCCAAATCCTCAAAGAGCATCCTGTCGAGAACACCCAGCTCCAGAACGAGAAGCTCTACCTCTACTACCTCCAGAACGGCCGCGATATGTACGTCGATCAAGAGCTGGACATCAACCGCCTCTCCGATTACGATGTCGATCATATCGTCCCTCAGTCCTTCCTGAAGGATGATTCCATCGATAACAAGGTCCTCACCCGCTCCGATAAGAACCGCGGCAAGTCCGATAACGTCCCTTCCGAAGAGGTCGTCAAGAAGATGAAGAACTACTGGCGCCAGCTCCTCAACGCCAAGCTCATCACCCAGCGCAAGTTCGATAACCTCACCAAGGCCGAGCGCGGTGGCCTCTCCGAGCTGGATAAGGCCGGCTTCATCAAGCGCCAGCTCGTCGAAACCCGCCAGATCACCAAGCACGTCGCCCAAATCCTCGATTCCCGCATGAACACCAAGTACGATGAGAACGATAAGCTCATCCGCGAAGTCAAGGTCATCACCCTCAAGTCCAAGCTCGTCAGCGATTTCCGCAAGGATTTCCAGTTCTACAAGGTCCGCGAGATCAACAACTACCATCATGCCCATGATGCCTACCTCAACGCCGTCGTCGGCACCGCCCTCATCAAGAAGTACCCCAAGCTCGAATCCGAGTTCGTCTACGGTGATTACAAGGTCTACGATGTCCGCAAGATGATCGCCAAGTCCGAGCAAGAGATCGGCAAGGCTACCGCCAAGTACTTCTTCTACTCCAACATCATGAATTTCTTCAAGACCGAAATCACCCTCGCCAACGGCGAAATCCGCAAGCGCCCTCTCATCGAGACTAACGGCGAGACTGGCGAGATCGTCTGGGATAAGGGCCGCGATTTCGCCACCGTCCGCAAGGTCCTCTCCATGCCTCAGGTCAACATCGTCAAGAAAACCGAGGTCCAGACCGGCGGCTTCTCCAAAGAGTCCATCCTCCCCAAGCGCAACTCCGATAAGCTGATCGCCCGCAAGAAGGATTGGGACCCTAAGAAGTACGGCGGCTTCGATTCCCCTACCGTCGCCTACTCCGTCCTCGTCGTCGCCAAGGTCGAGAAGGGCAAGTCCAAGAAGCTCAAGTCCGTCAAAGAGCTGCTCGGCATCACTATTATGGAACGCTCCAGCTTCGAGAAGAACCCTATCGATTTCCTTGAGGCCAAGGGCTACAAAGAGGTCAAGAAGGACCTCATCATCAAGCTCCCCAAGTACTCCCTGTTCGAGCTTGAGAACGGCCGCAAGCGCATGCTCGCCTCCGCCGGTGAGCTTCAGAAGGGCAACGAGCTGGCCCTGCCTTCCAAGTACGTCAACTTCCTCTACCTCGCCTCCCATTACGAGAAGCTCAAGGGCTCCCCTGAGGATAACGAGCAGAAGCAGCTGTTCGTCGAGCAGCATAAGCACTACCTCGATGAGATCATCGAGCAGATCAGCGAGTTCTCCAAGCGCGTCATCCTCGCCGATGCCAACCTCGATAAGGTCCTGTCCGCCTACAACAAGCACCGCGATAAGCCTATCCGCGAGCAGGCCGAGAACATCATCCATCTCTTCACCCTCACCAACCTCGGTGCCCCTGCCGCCTTCAAGTACTTCGATACCACCATCGATCGCAAGCGCTACACCTCCACCAAAGAGGTCCTGGACGCCACCCTCATCCATCAGTCCATCACCGGCCTCTACGAAACCCGCATCGATCTCTCCCAGCTCGGCGGCGACGGCGGTGGCGGATCCGGCGGTGGCGGTTCCGGCGGTGGCGGTTCCATGGTGAGCAAGGGCGAGGAGCTGTTCACCGGGGTGGTGCCCATCCTGGTCGAGCTGGACGGCGACGTAAACGGCCACAAGTTCAGCGTGTCCGGCGAGGGCGAGGGCGATGCCACCTACGGCAAGCTGACCCTGAAGTTCATCTGCACCACCGGCAAGCTGCCCGTGCCCTGGCCCACCCTCGTGACCACCTTCACCTACGGCGTGCAGTGCTTCAGCCGCTACCCCGACCACATGAAGCAGCACGACTTCTTCAAGTCCGCCATGCCCGAAGGCTACGTCCAGGAGCGCACCATCTTCTTCAAGGACGACGGCAACTACAAGACCCGCGCCGAGGTGAAGTTCGAGGGCGACACCCTGGTGAACCGCATCGAGCTGAAGGGCATCGACTTCAAGGAGGACGGCAACATCCTGGGGCACAAGCTGGAGTACAACTACAACAGCCACAACGTCTATATCATGGCCGACAAGCAGAAGAACGGCATCAAGGTGAACTTCAAGATCCGCCACAACATCGAGGACGGCAGCGTGCAGCTCGCCGACCACTACCAGCAGAACACCCCCATCGGCGACGGCCCCGTGCTGCTGCCCGACAACCACTACCTGAGCACCCAGTCCGCCCTGAGCAAAGACCCCAACGAGAAGCGCGATCACATGGTCCTGCTGGAGTTCGTGACCGCCGCCGGGATCACTCTCGGCATGGACGAGCTGTACAAGAAGCGCCCCGCCGCCACCAAGAAGGCCGGCCAGGCTAAGAAGAAGAAGTGA |

Note: Black letters indicate the codon-optimized *cas9* gene. Purple letter indicate the NLS sequences of SV40 at 5’-termini and nucleoplasmin at 3’-termini. Green letters indicate the *gfp* (S65T) gene from pMF272. Orange letters indicate the (G_4_S)_3_ linker sequence.

## Table S5 DNA sequences of sgRNA constructs used in this study

| sgRNA constructs | DNA Sequence (5’ to 3’) |
| --- | --- |
| sgRNA1.0  (P*hU6*:sgRNA) | GAGGGCCTATTTCCCATGATTCCTTCATATTTGCATATACGATACAAGGCTGTTAGAGAGATAATTGGAATTAATTTGACTGTAAACACAAAGATATTAGTACAAAATACGTGACGTAGAAAGTAATAATTTCTTGGGTAGTTTGCAGTTTTAAAATTATGTTTTAAAATGGACTATCATATGCTTACCGTAACTTGAAAGTATTTCGATTTCTTGGCTTTATATATCTTGTGGAAAGGACGAAAGGACGAAAGCACCggGTCTTCgaGAAGACCTGTTTTAGAGCTAGAAATAGCAAGTTAAAATAAGGCTAGTCCGTTATCAACTTGAAAAAGTGGCACCGAGTCGGTGCTTTTTT |
| sgRNA1.1  (P*hU6*:sgRNA-albA) | GAGGGCCTATTTCCCATGATTCCTTCATATTTGCATATACGATACAAGGCTGTTAGAGAGATAATTGGAATTAATTTGACTGTAAACACAAAGATATTAGTACAAAATACGTGACGTAGAAAGTAATAATTTCTTGGGTAGTTTGCAGTTTTAAAATTATGTTTTAAAATGGACTATCATATGCTTACCGTAACTTGAAAGTATTTCGATTTCTTGGCTTTATATATCTTGTGGAAAGGACGAAAGGACGAAAGCACCAGTGGGATCTCAAGAACTACGTTTTAGAGCTAGAAATAGCAAGTTAAAATAAGGCTAGTCCGTTATCAACTTGAAAAAGTGGCACCGAGTCGGTGCTTTTTT |
| sgRNA2.0 (P*yU6*:sgRNA) | GATCGATAATTCTCCATAATAGTTCTGTTATTTATAATCTCCAGCACTAATAAATGCTATACGTATATTTGTACACAATATAATTTCAGAATTTATATTGCTACCATGACTGTCTGAGAATTGGGGGAATAACTTGATAATTGTTGGGATTCCATTGTTCGTAAACGCAATAATATTAGGTATATAGAAGATACTAAATGTTCTCTCCGAGGATATAGGAATGCTCACAATGGAATCGATATATTTCTACATAATAGTATTGAGATTATTCCTCTTTTAGTTTTATATAATTCATTATCCTATTACATTATCAATCCTTGCATTTCAGCTTCCATTAGACTTAATGACTGTTTCTCAATTTTTATGTCATCTTCCTGGACCTCATGTGATACTATACCAGTAGCATGAATACTACTGAATCGATGATACTTTAGAGTTTCATTGCAACAGTTTCAACACAGCCTGGCATGAACAGTGGTAAAAGTATTTCGTCCACTATTTTCGGCTACTATAAATAAATGTTTTTTTCGCAACTATGTGCACCggGTCTTCgaGAAGACCTGTTTTAGAGCTAGAAATAGCAAGTTAAAATAAGGCTAGTCCGTTATCAACTTGAAAAAGTGGCACCGAGTCGGTGCTTTTTT |
| sgRNA2.1 (P*yU6*-sgRNA-albA) | CCCAAGCTTGATCGATAATTCGCCATGGCGGCCGCGGGAATTCGATTCCCAAGCTTGATCGATAATTCTCCATCCTGCTCAGACCTCACCACCCCGGAGCAGCCGCGTCGTAGCAACCATCAGCTTCCATTAAGACTAATGACTGTTTCTCAATTCTTTATGTCATCTTTCCTGAGGACCGGCCTAGAGCGGAAACACATGTGCAGATCCCTTCATGTGATACTATACCAGTAGCATGAATACCTACTGGGCATTGGCGGTTTAATCGATGATACTTTAGAGTTTCATGGCAATCAGCAACAGTTTCAACAACCGCCGGTATAAGGCATGAACAGTGGTCAAGTCCGCTGTAAAGTATTTCGTCCTACTATCTCGGCTACTATAAATAAATGTTTTTTCGATCTATGTGCACCAGTGGGATCTCAAGAACTACGTTTTAGAGCTAGAAATAGCAAGTTAAAATAAGGCTAGTCCGTTATCAACTTGAAAAAGTGGCACCGAGTCGGTGCTTTTTT |
| sgRNA3.0 (P*anU6*:sgRNA) | CCCAAGCTTGATCGATAATTCGCCATGGCGGCCGCGGGAATTCGATTCCCAAGCTTGATCGATAATTCTCCATCCTGCTCAGACCTCACCACCCCGGAGCAGCCGCGTCGTAGCAACCATCAGCTTCCATTAAGACTAATGACTGTTTCTCAATTCTTTATGTCATCTTTCCTGAGGACCGGCCTAGAGCGGAAACACATGTGCAGATCCCTTCATGTGATACTATACCAGTAGCATGAATACCTACTGGGCATTGGCGGTTTAATCGATGATACTTTAGAGTTTCATGGCAATCAGCAACAGTTTCAACAACCGCCGGTATAAGGCATGAACAGTGGTCAAGTCCGCTGTAAAGTATTTCGTCCTACTATCTCGGCTACTATAAATAAATGTTTTTTCGATCTATGTGCACCggGTCTTCgaGAAGACCTGTTTTAGAGCTAGAAATAGCAAGTTAAAATAAGGCTAGTCCGTTATCAACTTGAAAAAGTGGCACCGAGTCGGTGCTTTTTT |
| sgRNA3.1 (P*anU6*-sgRNA-albA1) | CCCAAGCTTGATCGATAATTCGCCATGGCGGCCGCGGGAATTCGATTCCCAAGCTTGATCGATAATTCTCCATCCTGCTCAGACCTCACCACCCCGGAGCAGCCGCGTCGTAGCAACCATCAGCTTCCATTAAGACTAATGACTGTTTCTCAATTCTTTATGTCATCTTTCCTGAGGACCGGCCTAGAGCGGAAACACATGTGCAGATCCCTTCATGTGATACTATACCAGTAGCATGAATACCTACTGGGCATTGGCGGTTTAATCGATGATACTTTAGAGTTTCATGGCAATCAGCAACAGTTTCAACAACCGCCGGTATAAGGCATGAACAGTGGTCAAGTCCGCTGTAAAGTATTTCGTCCTACTATCTCGGCTACTATAAATAAATGTTTTTTCGATCTATGTGCACCAGTGGGATCTCAAGAACTACGTTTTAGAGCTAGAAATAGCAAGTTAAAATAAGGCTAGTCCGTTATCAACTTGAAAAAGTGGCACCGAGTCGGTGCTTTTTT |

Note: Green letters indicate the promoter region for sgRNA expression. Orange letters indicate the transcription start of *U6* promoters. Blue letters indicate the sgRNA scaffold. Blue underlined letters indicate *Bbs*I restriction sites. Red letters indicate the terminator of *RNU6* gene. Red underlined letters indicate genetic targets.

## Table S6 DNA sequences of donor DNA used in this study

| Donor DNA | DNA Sequence (5’ to 3’) |
| --- | --- |
| MHi-albA-hph | CCTCCGCCTCCCAGCCTACAAGTGGGATCTCAAGAACTACgacgttaactgatattgaaggagcactttttgggcttggctggagctagtggaggtcaacaatgaatgcctattttggtttagtcgtccaggcggtgagcacaaaatttgtgtcgtttgacaagatggttcatttaggcaactggtcagatcagccccacttgtagcagtagcggcggcgctcgaagtgtgactcttattagcagacaggaacgaggacattattatcatctgctgcttggtgcacgataacttggtgcgtttgtcaagcaaggtaagtgaacgacccggtcataccttcttaagttcgcccttcctccctttatttcagattcaatctgacttacctattctacccaagcatcgatatgaaaaagcctgaactcaccgcgacgtctgtcgagaagtttctgatcgaaaagttcgacagcgtctccgacctgatgcagctctcggagggcgaagaatctcgtgctttcagcttcgatgtaggagggcgtggatatgtcctgcgggtaaatagctgcgccgatggtttctacaaagatcgttatgtttatcggcactttgcatcggccgcgctcccgattccggaagtgcttgacattggggaattcagcgagagcctgacctattgcatctcccgccgtgcacagggtgtcacgttgcaagacctgcctgaaaccgaactgcccgctgttctgcagccggtcgcggaggccatggatgcgatcgctgcggccgatcttagccagacgagcgggttcggcccattcggaccgcaaggaatcggtcaatacactacatggcgtgatttcatatgcgcgattgctgatccccatgtgtatcactggcaaactgtgatggacgacaccgtcagtgcgtccgtcgcgcaggctctcgatgagctgatgctttgggccgaggactgccccgaagtccggcacctcgtgcacgcggatttcggctccaacaatgtcctgacggacaatggccgcataacagcggtcattgactggagcgaggcgatgttcggggattcccaatacgaggtcgccaacatcttcttctggaggccgtggttggcttgtatggagcagcagacgcgctacttcgagcggaggcatccggagcttgcaggatcgccgcggctccgggcgtatatgctccgcattggtcttgaccaactctatcagagcttggttgacggcaatttcgatgatgcagcttgggcgcagggtcgatgcgacgcaatcgtccgatccggagccgggactgtcgggcgtacacaaatcgcccgcagaagcgcggccgtctggaccgatggctgtgtagaagtactcgccgatagtggaaaccgacgccccagcactcgtccgagggcaaaggaatagagtagatgccgaccggatcgatccacttaacgttactgaaatcatcaaacagcttgacgaatctggatataagatcgttggtgtcgatgtcagctccggagttgagacaaatggtgttcaggatctcgataagatacgttcatttgtccaagcagcaaagagtgccttctagtgatttaatagctccatgtcaacaagaataaaacgcgtttcgggtttacctcttccagatacagctcatctgcaatgcattaatgcattggacctcgcaaccctagtacgcccttcaggctccggcgaagcagaagaatagcttagcagagtctattttcattttcgggagacgagatcaagcagatcaacggtcgtcaagagacctacgagactgaggaatccgctcttggctccacgcgactatatatttgtctctaattgtactttgacatgctcctcttctttactctgatagcttgactatgaaaattccgtcaccagcccctgggttATTCCCTATACCAACAACTTCTGCCTGAGCAAGGGCGCTC |

Note: Blue letters indicate the homogenous arms located at the 5’ and 3’ flanking region of the genetic target sites. Black lowercase letters indicate the selection marker cassettes.
